# Disruption of insulin signalling preserves bioenergetic competence of mitochondria in ageing *Caenorhabditis elegans*

**DOI:** 10.1186/1741-7007-8-91

**Published:** 2010-06-28

**Authors:** Kristel Brys, Natascha Castelein, Filip Matthijssens, Jacques R Vanfleteren, Bart P Braeckman

**Affiliations:** 1 Department of Biology, Ghent University, K L Ledeganckstraat 35, Ghent B-9000, Belgium

## Abstract

**Background:**

The gene *daf-2 *encodes the single insulin/insulin growth factor-1-like receptor of *Caenorhabditis elegans*. The reduction-of-function allele *e1370 *induces several metabolic alterations and doubles lifespan.

**Results:**

We found that the *e1370 *mutation alters aerobic energy production substantially. In wild-type worms the abundance of key mitochondrial proteins declines with age, accompanied by a dramatic decrease in energy production, although the mitochondrial mass, inferred from the mitochondrial DNA copy number, remains unaltered. In contrast, the age-dependent decrease of both key mitochondrial proteins and bioenergetic competence is considerably attenuated in *daf-2(e1370) *adult animals. The increase in *daf-2(e1370) *mitochondrial competence is associated with a higher membrane potential and increased reactive oxygen species production, but with little damage to mitochondrial protein or DNA. Together these results point to a higher energetic efficiency of *daf-2(e1370) *animals.

**Conclusions:**

We conclude that low *daf-2 *function alters the overall rate of ageing by a yet unidentified mechanism with an indirect protective effect on mitochondrial function.

## Background

The lifespan of *Caenorhabditis elegans *is regulated by multiple signalling pathways that converge on a battery of downstream target genes. Among these, insulin/insulin growth factor-1-like (IGF) signalling (IIS) is currently best understood. Activated insulin/IGF-1-like receptor encoded by the gene *daf-2 *triggers downstream kinases to phosphorylate a FOXO transcription factor encoded by *daf-16*. Phosphorylated DAF-16 protein is sequestered in the cytoplasm and inactive. Reduction of IIS in the absence of ligand or via reduction- or loss-of-function mutation in the *daf-2 *gene relocates DAF-16 to the nucleus and triggers a genetic program for lifespan extension [1-7].

Mutation in *daf-2 *also enhances resistance to oxidative and thermal stress, most likely by activating several superoxide dismutase and heat-inducible genes [8-11]. The concurrent features of longevity and resistance to oxidative stress have been interpreted as supporting the oxidative stress theory of ageing which proposes a central role for oxygen free radicals and derived reactive oxygen species (ROS) in causing the ageing process [12-17]. Mitochondria convert approximately 0.1%-0.3% of the consumed oxygen to superoxide which can further react to generate other ROS [18,19]. Hence, a widely held view is that ageing initiates in, and spreads from, the mitochondrial compartment [18,20-22].

The *e1370 *mutant allele of *daf-2 *conveys several other phenotypic traits to the animals, including a slender adult body, reduced brood size, resistance to hypoxia and enhanced autophagy [1,23]. *daf-2(e1370) *animals have an altered metabolism that is partly reminiscent of the dauer stage, a developmental stage that does not feed and is adapted for long-term survival. Dauers are hypometabolic and rely, in part, on a shift to anaerobic energy metabolism [24-26]. *daf-2(e1370) *adult animals consume substantially less food and they dissipate less CO_2 _relative to wild-type controls on a per animal base [27], but their mass-specific oxygen consumption rates suggest that they are not hypometabolic [28].

Unexpectedly, the latter experiments also demonstrated that *daf-2(e1370) *worms dissipate less heat than wild-type animals per mole of oxygen utilized. This difference was manifested by a considerable reduction of the calorimetric to respirometric (C/R) ratio, possibly pointing to a higher efficiency of mitochondrial metabolism (hereafter called 'energetic efficiency') of *daf-2(e1370) *animals. In order to address the potential link between energy metabolism, ROS production and oxidative damage and lifespan, we have examined energy production in intact worms and in isolated mitochondria. We found that the age-dependent decrease of bioenergetic competence is considerably attenuated in *daf-2(e1370) *adult animals. We also found that the higher mitochondrial competence was associated with increased ROS production, but with little, if any, damage to mitochondrial protein or DNA. The low C/R ratio observed in live *daf-2(e1370) *worms was not recapitulated in isolated mitochondria, suggesting that other targets of IIS act in concert with the mitochondria to control organismal metabolic rate.

## Results

### IIS alters aerobic energy production

**Figure 1:**
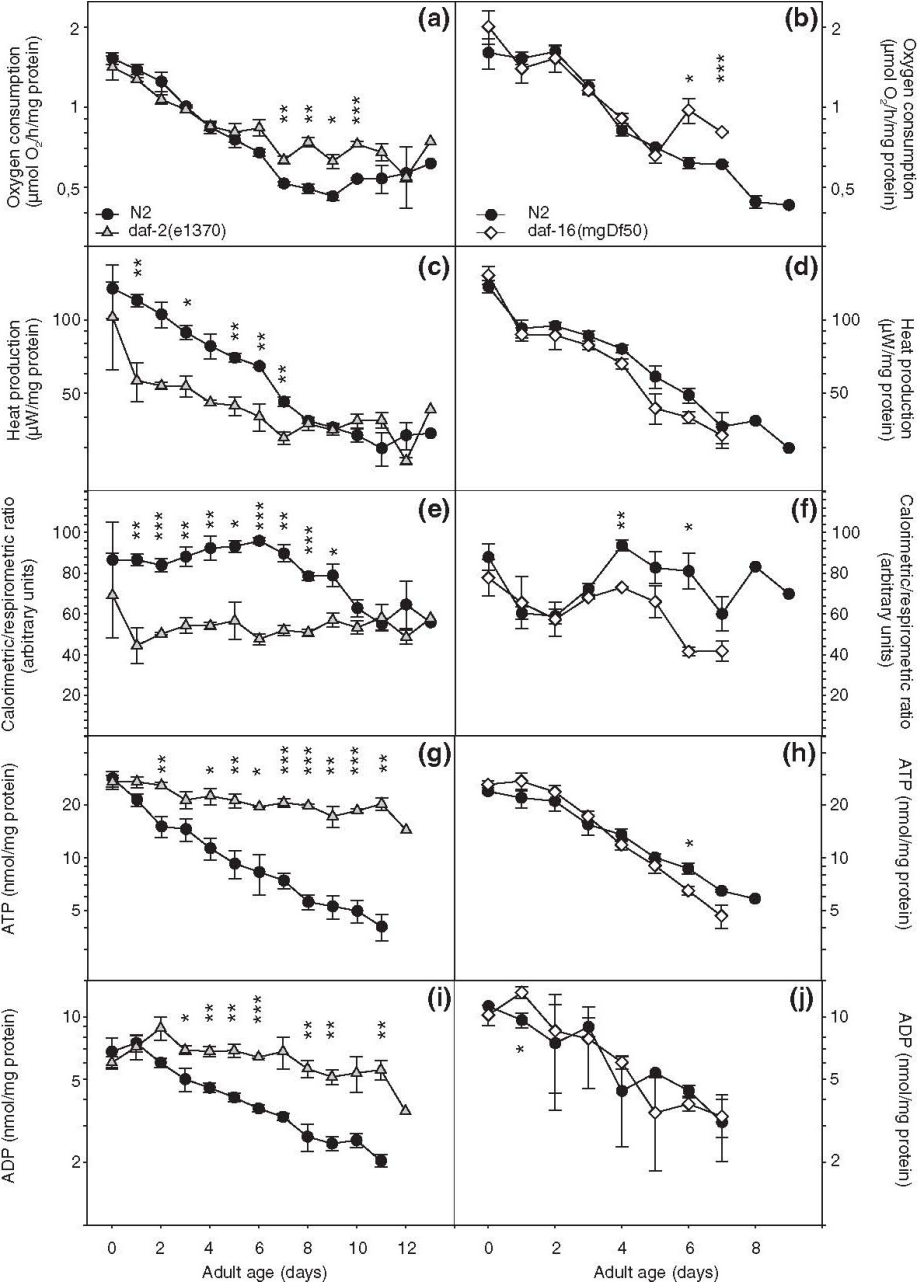
**Ins/IGF-1 signalling controls age-related changes in aerobic energy production**. Left panels: wild-type (WT) versus *daf-2(e1370)*, right panels: WT versus *daf-16(mgDf50)*. (a-b) Respiration rate. (c-d) Metabolic heat production. (e-f) Calorimetric to respirometric ratio. (g-h) Adenosine triphosphate content. (i-j) Adenosine diphosphate content. Displayed values are means ± standard error of mean for three replicate cultures; *, *P *< 0.05, **, *P *< 0.01 and ***, *P *< 0.001.

We obtained respiration and heat production rates from wild-type (N2) worms, *daf-2(e1370) *and *daf-16(mgDf50) *animals. *Df50 *is a molecular null allele denoting a large deficiency that deletes nearly the entire *daf-16 *coding region [6]. This mutation is expected to suppress all Daf-2 phenotypes that rely on intact DAF-16. Respiration declined with age in all three strains. The rate of decrease was smaller in *daf-2(e1370) *animals [*P*_age*strain _(d0-d7) = 0.0002], but overall, respiration rates were grossly similar in all three strains (Figure 1a and 1b). Heat dissipation also decreased with age in N2 and *daf-16(mgDf50) *but was markedly lower in *daf-2(e1370) *animals (Figure 1c and 1d). Since the C/R ratio provides an indication of catabolic efficiency [29], these results indicate that the efficiency of aerobic energy production is upregulated in *daf-2(e1370) *animals during the first 7-9 days of their adult lifespan (Figure 1e and 1f). Interestingly, loss of *daf-16 *function also caused lower C/R ratios than wild-type after the third day of adult life. A similar observation was previously reported for the reduction-of-function allele *daf-16(m26) *[30]. Next we asked if this putative upregulation of catabolic efficiency in *daf-2(e1370) *would be observed in the standing levels of adenosine triphosphate (ATP) and adenosine diphosphate (ADP) and we found that this was indeed the case (Figure 1g and 1i) [*daf-2 *versus N2, both ATP and ADP: *P*_age*strain _< 0.0001]. Thus, unlike wild-type (WT) and *daf-16(0) *worms, *daf-2(e1370) *animals are able to attenuate the age-specific depletion of the instantly utilizable energy source ATP.

### The age-specific decrease in energy production is not caused by systematic loss of mitochondrial genome copy number

**Figure 2:**
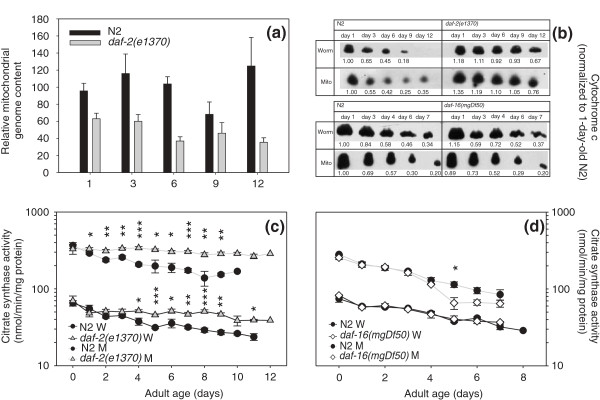
**Effect of age on mitochondrial genome and cytochrome *c *content and citrate synthase activity**. (a) Age-specific mitochondrial genome content of wild-type and *daf-2(e1370) *animals. Three mitochondrial genes were quantified; the results were normalized to obtain the relative mitochondrial genome content per strain and age cohort. The error bars indicate ± standard error of mean for three mitochondrial genes and three replicate ageing cohorts. (b) Western blots showing age-related changes of cytochrome *c *abundance in crude worm extract and isolated mitochondria. Results from one representative experiment are shown. The numeric values below each spot denote the abundance of cytochrome *c *in that spot normalized to the corresponding spot from 1-day-old wild-type (WT) adults. (c-d) Activity levels of citrate synthase in crude worm extract (indicated as 'W') and in isolated mitochondria (indicated as 'M'). Left panel: WT versus *daf-2(e1370)*, right panel: WT versus *daf-16(mgDf50)*. Data represent means ± standard error of mean (bars) for at least three replicate cultures; *, *P *< 0.05, **, *P *< 0.01, ***, *P *< 0.001.

Having established that whole-worm energy production declines dramatically with age, we next asked whether this could be caused by age-related loss of mitochondria. We used quantitative real-time polymerase chain reaction (qPCR) to assay the copies of mtDNA in *daf-2(e1370) *and WT animals. We quantitated three mitochondrial genes in staged worms from three independent replicate cultures in order to enforce the robustness of the observations. Counter to our expectation, we did not detect any age-related changes in mitochondrial DNA (mtDNA) content [*P*_age _not significant (NS); Figure 2a]. Interestingly, wild-type animals had about double the number of mtDNA copies compared to *daf-2(e1370) *worms, with *P *values between strains bordering on significance (*P*_strain _= 0.0585); this difference is likely caused by differences in germline proliferation.

### The *daf-2(e1370) *allele attenuates the age-specific decline in abundance of key mitochondrial proteins

The isolation of mitochondria from nematode tissue requires harsh treatments to break the tough cuticle. If old worms contain more fragile mitochondria, the isolation process might, by itself, yield a larger portion of damaged mitochondria. In order to ascertain that mitochondrial isolation does not lead to disproportionate amounts of damaged organelles with progressing age and strain differences, we compared the activity level of citrate synthase, a key citric acid cycle enzyme of the matrix and the abundance of cytochrome *c*, an essential component of the electron transport chain which is present in the intermembrane space. We reasoned that, if the relative abundance of these proteins in isolated mitochondria and in whole worm extracts is identical, this would indicate that the preparation procedure caused no harm to the mitochondria or, at least, that possible damage to the mitochondrial inner or outer membrane inflicted by the isolation process was proportionate at all ages.

Western blots showed that cytochrome *c *protein levels declined with age in all three strains and at similar rates in both mitochondrial preparations and whole worm extracts. Remarkably, this decline was much slower in the long-lived *daf-2(e1370) *indicating that these mutant animals can attenuate age-dependent reduction in cytochrome *c *content. As this effect was also seen in whole worm extracts, it is not due to higher resilience of the *daf-2 *mitochondrial outer membrane to disruption during isolation (Figure 2b). Similarly, mitochondrial preparations and whole worm extract yielded identical age-specific activity profiles of citrate synthase. Much like cytochrome *c *content, citrate synthase activity declined more gradually with age in *daf-2(e1370) *than in WT and *daf-16(0) *animals (Figure 2c,d; *daf-2 *versus N2: *p*_age*strain _in worms = 0.0074; in mitochondria = 0.0485; *daf-16 *versus N2, for both worm and mitochondria: *P *_strain _= NS, *P*_age _< 0.0001). These results dispel the notion that mitochondrial preparations from wild-type worms might contain a higher proportion of disrupted organelles and concomitant loss of mitochondrial proteins.

Other mitochondrial proteins also show alterations with age. We quantified the abundance of the complex I NDUFS3 subunit, the pyruvate dehydrogenase subunit E1α, complex IV subunit I, complex V subunits α and ß and adenine nucleotide transferase on Western blots of whole worm extracts. For the Complex I NDUFS3 subunit, pyruvate dehydrogenase subunit E1α, adenine nucleotide transferase and complex V subunit α, a clear decrease in protein content with age was observed in WT worms, whereas only minor decreases were detected in long-lived *daf-2(e1370) *animals. For complex V subunit ß, we observed a slight decrease with age in both strains but the *daf-2 *signal was generally higher than that of WT. Only complex IV subunit I content did not decrease with increasing age (Additional File 1: Figure S1).

### The *daf-2(e1370) allele *ensures high bioenergetic competence throughout the adult life of the animals

Mitochondria can shift between several energetic states depending on the presence of combustible substrate and ADP. Freshly purified mitochondria lack sufficient amounts of both and consume very little amounts of oxygen, called state 1 respiration. The addition of metabolizable substrate (state 2 respiration) is not helpful as long as there is no ADP to unlock complex V and permit protons to flow into the mitochondrial matrix. The addition of sufficient amounts of substrate and ADP maximizes proton flow through complex V and, consequently, electron transport and the reduction of oxygen to water at complex IV (state 3 respiration), coupled to the conversion of ADP to ATP (oxidative phosphorylation). When ADP is depleted, the mitochondria return to the resting state 4 respiration.

**Figure 3:**
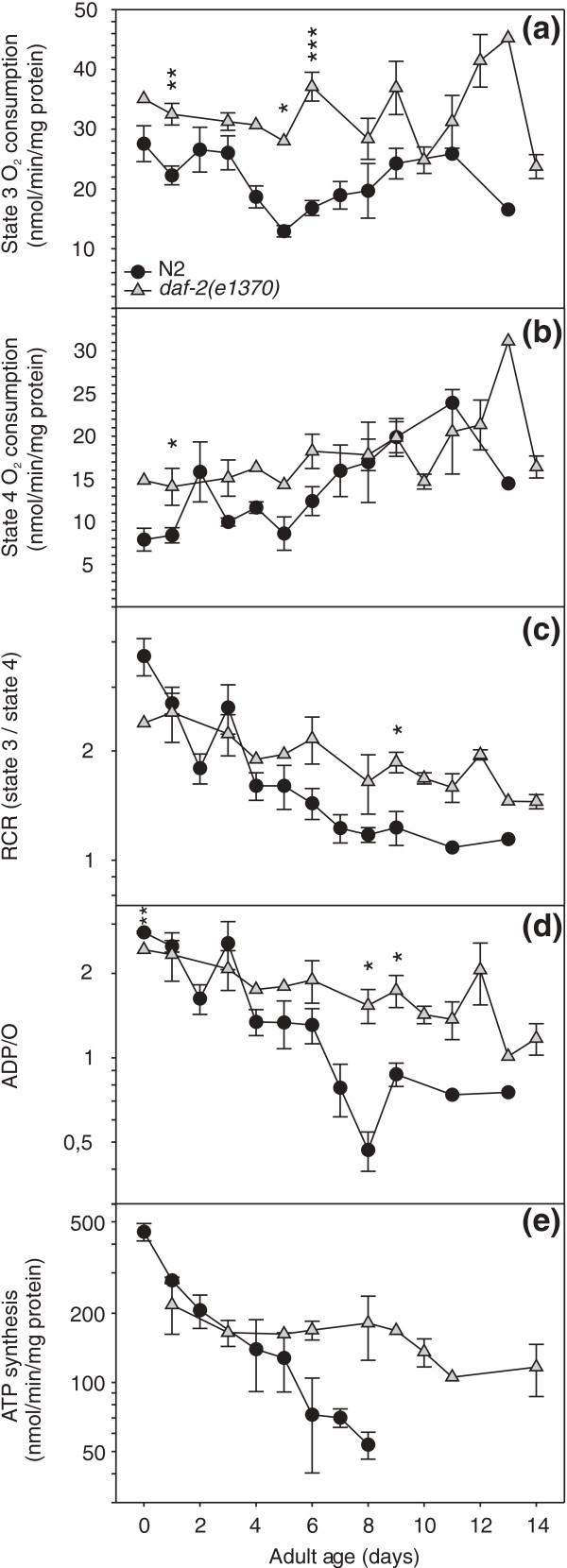
**The *daf-2(e1370) *allele preserves mitochondrial bioenergetic competence throughout the adult life trajectory**. (a) State 3 oxygen consumption. (b) State 4 oxygen consumption. (c) Respiratory control ratio. (d) Adenosine diphosphate (ADP)/O ratio. (e) ATP synthesis by isolated mitochondria.

We found that increasing age had little, if any, effect on state 3 respiration. ADP- stimulated oxygen consumption was higher [*P*_strain _= 0.0002] in *daf-2(e1370) *mitochondria (Figure 3a). In both N2 and *daf-2(e1370)*, state 3 respiration remained stable [*P*_age _= NS] over the entire life time studied (Figure 3a), whereas state 4 respiration increased with age very gradually (*P*_age _= 0.0002 and *P*_strain _= 0.0039; Figure 3b). The limitation placed on electron transport by the chemiosmotic gradient, or respiratory control, can be derived from these data. The ratio of substrate driven oxygen consumption in the presence of ADP (state 3) to that in its absence (state 4), or respiratory control ratio (RCR) decreased with age in both strains, but faster in N2 (*P*_age*strain _< 0.0001; Figure 3c). The passage of electrons through the proton translocating complexes I, III and IV is associated with the release of free energy that is recovered when protons flow back inside at complex V and ADP is converted to ATP. Based on the change in free energy under standard conditions, the theoretical ratio of ADP molecules that can be phosphorylated per atom oxygen that is reduced to water, or ADP/O ratio, is > 7 [31], but maximum attainable ratios are less because of various losses in this process and are ~three for oxidation of NADH by complex I. We observed a gradual age-related decline of ADP/O in *daf-2(e1370) *and a steeper one in N2 (*P*_age*strain _= 0.0001; Figure 3d).

Next we measured the rate of ATP synthesis by isolated mitochondria of both strains in the presence of non-limiting supply of substrate and ADP. The results reflected the ADP/O profiles. Complex-I-dependent ATP synthesis capacity of *daf-2(e1370) *mitochondria was hardly affected by the ageing process, whereas a gradual decline was observed for mitochondria prepared from WT animals (*P*_age*strain _= 0.0222; Figure 3e). Similar ATP synthesis results were obtained when the mitochondria were fuelled with complex II substrate (results not shown).

### Unlike the live animals, *daf-2(e1370) *mitochondria show no dramatic reduction in C/R ratio

A portion of the protons pumped into the intermembrane space by ETC complexes I, III and IV is not used to drive ADP phosphorylation by complex V, but leaks back to the matrix and represents a loss of energy as heat. Since heat released by live *daf-2(e1370) *animals was abnormally low relative to WT worms, we asked whether isolated mitochondria would yield similar results. A disadvantage of our thermal activity monitoring method is that it requires prolonged (~40 min) temperature equilibration of the samples in the instrument prior to effective data collection (~10 min). We observed that the oxygen consumption by *daf-2(e1370) *mitochondria was reduced by 15% at the end of the experiment compared to the initial respiration rate (results not shown). Remarkably, WT mitochondria lost very little activity under these conditions. To minimize experimental bias, we only used the respiration rates measured after completion of heat measurement to obtain the C/R ratio. Mitochondria respiring in state 3 fuelled by non-limiting amounts of ADP and complex I substrate were used and this experiment was repeated eight times. Overall, we found no significant difference in C/R between the mutant and wild-type mitochondria. (Additional File 2: Figure S2).

### The *daf-2(e1370) *allele causes a higher mitochondrial membrane potential and enhanced respiratory capacity

**Figure 4:**
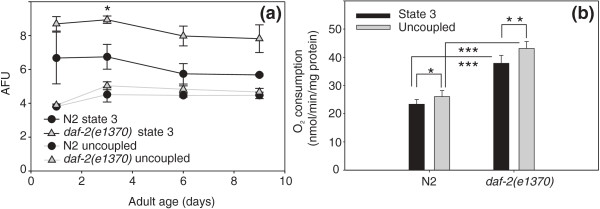
**The *daf-2(e1370) *allele causes a higher mitochondrial membrane potential and enhanced respiratory capacity**. (a) Increased accumulation of the fluorescent probe DASPMI inside energized *daf-2(e1370) *mitochondria. Fluorescence intensity of DASPMI in energized and uncoupled isolated mitochondria of wild-type (WT) and *daf-2(e1370)*. Fluorescence emission over the 2 min interval was averaged for each energetic state. Displayed values are means ± standard error of mean for three replicate cultures. The increase of DASPMI fluorescence is proportional to the amount of dye taken up by the mitochondria which itself is proportional to the membrane potential (Mewes and Rafael [33]). (b) Higher oxygen consumption in the presence of adenosine diphosphate or uncoupler by *daf-2(e1370) *mitochondria. The assay was performed on two replicate cultures; since no age-dependent differences were noticed, mitochondrial respiration rates were averaged per strain over a 9-day time span. Paired *t*-tests were performed in within-strain comparisons, unpaired *t*-tests were performed in between-strain comparisons; * *P *< 0.05, ** *P *< 0.01 and *** *P *< 0.001.

We considered the possibility that microcalorimetry of isolated mitochondria lacked the necessary resolving power to detect small differences in mitochondrial heat dissipation. Since mitochondrial heat production is inversely proportional with electron transport chain efficiency, we asked if *daf-2(e1370) *mitochondria operate at a higher membrane potential. We used the cationic fluorescent dye DASPMI to probe ΔΨ_mit _of mitochondria prepared from wild-type and *daf-2 *animals. The positively charged DASPMI ion readily penetrates the mitochondria and is distributed between the external space and the matrix compartment in accordance to the Nernst equation. The increase of emission of mitochondrial fluorescence is due to enhancement in quantum yield in the more proteinaceous and apolar microenvironment inside the mitochondria and is strictly linear up to ~3 nmol dye/mg mitochondrial protein [32,33]. We compared the fluorescence intensities of DASPMI in the activated OXPHOS state (state 3) and after addition of uncoupler (FCCP) which results in collapse of the membrane potential and equal distribution of the probe inside and outside the mitochondria (Figure 4a). The fluorescence intensities of uncoupled WT and *daf-2 *mitochondria were essentially identical (*P*_strain _NS) and invariant with age (*P*_age _NS). However, activated *daf-2 *mitochondria emitted more fluorescence than WT mitochondria (*P*_strain _0.0343) and the fluorescence intensities decreased with age in both strains (*P*_age _0.0157). We conclude that the membrane potential in fast-respiring mitochondria decreases with age and that *daf-2 *mitochondria most likely operate at higher ΔΨ_mit _values. Very recently, another study reported, using *in vivo *monitoring of the carbocyanine dye 'DiS-C_3_(3)' that knock-down of lifespan-limiting genes by mutation or RNAi results in a lower ΔΨ_mit _and a drop in the λ_max _of the emitted fluorescence [34]. These observations are difficult to interpret because they are heavily biased by strain-specific differences in feeding rate, and, of consequence, dye accumulation [35]. Lowered ΔΨ_mit _is also predicted to lower ROS production, as opposed to our measurements.

Since addition of uncoupler resulted in equal ΔΨ_mit _in both strains, we asked what the consequences of uncoupling would be on mitochondrial oxygen consumption. Addition of the uncoupler FCCP increased respiration to the level of maximum electron transport system capacity. The uncoupled rates were slightly higher than the respective state 3 rates, indicating that the mitochondria from both strains respired close to their maximum capacity. However, both uncoupled and state 3 rates were substantially higher for *daf-2(e1370) *relative to N2 indicating that this capacity is enhanced in *daf-2(e1370) *animals (Figure 4b).

### *daf-2(e1370) *mitochondria generate more H_2_O_2 _*in vitro *but do not reveal higher oxidative damage accumulation in live worms

**Figure 5:**
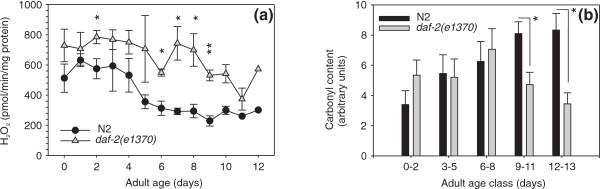
***daf-2(e1370) *mitochondria generate more H**_**2**_**O**_**2 **_**but do not reveal increased carbonyl load**. (a) H_2_O_2 _generation by isolated mitochondria from wild-type and *daf-2(e1370)*. Mitochondria were fuelled with pyruvate, malate and adenosine diphosphate. Cu/Zn SOD from erythrocytes was added to achieve maximal conversion of O_2 _to H_2_O_2_. Data represent means ± standard error of mean (SEM; bars) for mitochondria isolated from four replicate cultures; * *P *< 0.05 and ** *P *< 0.01. (b) Carbonyl content of mitochondrial protein derivatized with diphenylhydrazine and detected by Western blotting and a diphenylhydrazone specific antibody. Data represent means ± SEM. Six replicate cultures of each strain were grown but these were sampled at different time intervals, occasionally reducing the number of replicate samples for each time interval to 3; * *P *< 0.05.

In isolated mitochondria, the rate of ROS production is dependent on the mitochondrial membrane potential [36]. As reported, ΔΨ_mit _of energized mitochondria is higher in *daf-2(e1370) *than in the WT; this raises questions about levels of ROS production by these mitochondria. We measured H_2_O_2 _production in the presence of exogenous superoxide dismutase (SOD) to guarantee that all superoxide would be converted to H_2_O_2_. We found that H_2_O_2 _formation declined with age in all three strains, and that *daf-2(e1370) *mitochondria produced higher amounts at all ages tested (*P*_strain _= 0.0045; *P*_age _< 0.0001; Figure 5a), in line with their higher membrane potential. In contrast, WT animals and *daf-16(mgDf50) *mutants produced essentially identical amounts of H_2_O_2 _during their adult life trajectories (Additional File 3: Figure S3A).

We asked if this elevated production of ROS *in vitro *would be reflected in enhanced damage to mitochondrial macromolecules *in vivo*. Firstly, we assayed carbonyl groups on Western blots of mitochondrial samples. The extent of carbonylation was fairly identical in wild-type worms and *daf-2(e1370) *animals during the first week of their adult lives, but mitochondrial protein from old *daf-2(e1370) *animals carried substantially less carbonyl load relative to WT worms (Figure 5b). No differences were observed in the carbonyl load of mitochondrial protein prepared from wild-type and *daf-16(mgDf50) *animals (Additional File 3: Figure S3B).

ROS can also inflict damage to DNA. We monitored the occurrence of mitochondrial deletions with progressing age using long range nested PCR. For this experiment we used the long-lived double mutant strain *daf-2(e1370); glp-4(bn2) *and *daf-16(mgDf50) glp-4(bn2) *as a control. The *glp-4 *genetic background was chosen because it is defective in germline development at the restrictive temperature. In total we examined 624 *daf-2(e1370); glp-4(bn-2) *worms picked from 3-, 7-, 10-, 14- and 26-day-old adult cohorts, and 432 *daf-16(mgDf50) glp-4(bn2) *worms picked from 3-, 7- and 10-day-old adult cohorts. Faint bands representing curtailed fragments were observed incidentally, irrespective of strain or age. However, when the original DNA was assayed again these bands disappeared and novel bands incidentally arose, suggesting that these shortened fragments were generated artefactually during PCR amplification. Thus we found no evidence for the occurrence of deletions in mtDNA linked to strain differences or progressing age (results not shown).

## Discussion

### Aerobic energy production depends on the concerted action of mitochondrial performance and extra-mitochondrial regulation

We have previously noted that *daf-2(e1370) *animals consume similar amounts of O_2 _but dissipate substantially less heat than WT animals [28,30]. In the Houthoofd *et al*. study [30] we demonstrated that the mutant alleles *daf-2(e1370) *and *daf-16(m26) *did not produce the expected opposite changes in heat production and ATP content and that these phenotypes were not fully suppressed in the double mutant. Hence, we proposed that two pathways emanate from DAF-2, one which is predominant and DAF-16 independent, whereas the second one requires DAF-16 activity. Our present results agree with these previous findings.

Which mechanism(s) could be invoked to explain the uncoupling of respiration and heat dissipation? It has been speculated that a shift to glycolysis and fermentation along with aerobic respiration would generate energy in *daf-2(e1370) *adult animals, as is known for the dauer stage [37]. If so, this would rather increase the C/R ratio in *daf-2 *mutants since anaerobic metabolism produces heat without consuming oxygen. We assumed that reduction in C/R ratio reflects more efficient energy production since less energy is lost as heat [29] and expected to find enhanced coupling of ATP synthesis to the oxidation of NADH and FADH_2_. However, we could not reproduce the large difference in C/R ratios of live young adult *daf-2(e1370) *and WT animals by assaying their isolated mitochondria (Additional File 2: Figure S2). Also, the largest difference in worm C/R ratio was observed for a cohort of animals up to 7-9 days of adulthood, but this pattern was not reproduced by their mitochondrial ADP/O ratios. Conversely, we found that the ADP/O ratios for *daf-2(e1370) *mitochondria were generally higher than those obtained for WT worms when older animals were assayed, whereas the difference in worm C/R ratios between both strains faded away at advancing age (Figures 1e and 3d). We considered the possibility that the *daf-2(e1370) *mitochondria might contain more ATP synthase inhibitor protein, IF_1_. This protein has the capacity to inhibit the intrinsic F_1_-ATPase activity [38]. Since futile ATP hydrolysis is expected to generate heat, we reasoned that more IF_1 _might contribute to the reduced heat production in *daf-2(e1370) *animals. McElwee *et al*. [26] reported that one of both *C. elegans *IF_1 _encoding genes, *mai-1 *is expressed at a higher level in *daf-2*. However, *mai-1 *lacks a mitochondrial import signal (www.wormbase.org) casting doubt as to its mitochondrial action. We compared the expression of *mai-1 *and *mai-2 *(which has a mitochondrial import signal) in 2- and 8-day-old adults using qPCR and found no difference between the WT and *daf-2 *mutant strains nor between the age classes (results not shown). Thus, it appears that the aberrant C/R ratio of *daf-2(e1370) *animals cannot be merely ascribed to an intrinsic property of their mitochondria and extra-mitochondrial regulatory mechanisms must be considered. Uncoupling protein (UCP)-4 allows proton movement across the inner mitochondrial membrane and mutant animals lacking UCP-4 reportedly contain elevated ATP levels and are sensitive to cold stress [39]. The *ucp-4 *gene is expressed at equal levels in wild-type and *daf-2(e1370) *[26], yet the activity of the protein may be regulated differently in the mutant. Another possible regulatory mechanism is futile cycling of fructose-6-phosphate by phosphofructokinase and fructose bisphosphatase resulting in the net hydrolysis of ATP and thermogenesis [31]. This pathway is active in vertebrate species and has not yet been extensively studied in *C. elegans*. Phosphofructokinase is upregulated in *daf-2(e1370) *adult animals and both phosphofructokinase and fructose-1,6-bisphosphatase are upregulated in wild-type dauers [26,40]. Since dauers and *daf-2 *adults predominantly use fat stores for energy production, these changes most likely indicate that cells expressing glycolytic activity are different from those that are active in gluconeogenesis [40]. Thermogenesis by cycling of fructose-6-phosphate would involve opposing reactions of phosphofructokinase and fructose-1,6-bisphosphatase in the same tissue [31], is more likely under allosteric control and may be reduced in *daf-2 *adults relative to the WT animals.

### Age-dependent reduction of mitochondrial bioenergetic competence is attenuated in *daf-2(e1370) *animals

Conceivably, the dramatic reduction of energy production with age could be caused by systematic loss of mitochondria. We tested this hypothesis by assaying the mitochondrial genome content using a quantitative real time PCR approach and found that it was false. Next we investigated whether isolated mitochondria show a comparable age-dependent reduction in respiration rate. This was clearly not the case. state 3 respiration was essentially unaffected by age in both strains. State 3 respiration represents maximum performance under conditions of unlimited fuel supply, non-physiologically high oxygen concentration and absence of any cellular control. These results indicate that the ability of the mitochondria to reduce oxygen is not affected by the ageing process and that the decline of oxygen consumption and heat output by intact animals during the first week of adulthood is regulated by aspects of mitochondrial function not studied here or by extra-mitochondrial control. In contrast, the mitochondrial coupling efficiency, illustrated by the ADP/O ratio and ATP synthesis under state 3 conditions, and the dependence of respiration on the available ADP, illustrated by the RCR, declined with age in all three strains though more weakly in *daf-2(e1370) *mitochondria. These results suggest that the mitochondrial bioenergetic competence is bound to decline with age but that this decline is attenuated by mutation in *daf-2*, suggesting modulation by Ins/insuline-like growth factor (IGF-1) signalling.

How could the bioenergetic competence of the mitochondria be altered? We found that several components involved in mitochondrial function decreased with age both in N2 and *daf-2 *animals though faster in N2. That could explain the higher state 3 respiration rates measured for *daf-2 *but not the fairly constant state 3 rates measured over the life trajectories. One possible explanation is that the bioenergetic competence is largely dictated by a higher order structure of the ETC complexes. Bornhövd *et al*. [41] proposed a model of microdomain organization of OXPHOS (super)complexes in the mitochondrial inner membrane and they argued that disruption of these microdomains would affect metabolite/substrate channelling and/or efficient cooperation of these complexes, ultimately leading to a reduced flux through the respiratory chain and a lower membrane potential.

Our measurements of ADP/O, RCR, ATP synthesis and mitochondrial membrane potential in *daf-2 *worms versus wild type mitochondria are consistent with such a model. Most interestingly we can now explain the increased ROS production that is known to be positively correlated with the mitochondrial membrane potential [36,42].

### Mitochondrial ROS production does not limit the lifespan of *daf-2(e1370)*

Although we cannot prove that *daf-2(e1370) *mitochondria also generate more ROS *in vivo*, the expectation is that they do so. At first glance, this appears to be at odds with the common belief that a reduction of ROS underlies lifespan extension because it is predicted to slow down oxidative damage accrual (Reviewed by [43]). However, this mutant does show increased SOD and catalase activities and levels of reduced glutathione and resistance to oxidative stress [44-47]. Microarray analysis revealed that impairment of DAF-2 signalling enhanced DAF-16-dependent expression of *sod-3*, *hsp-16*, *gst-1*, *gst-4*, *mtl-1*, *ctl-1 *and *ctl-2 *[48,49]. Conceiveably, the activation of such a generalized defense could be mediated by a process called mitohormesis, where increases in mitochondrial ROS production cause an overcompensating induction of the antioxidant machinery resulting in extension of lifespan, as illustrated by Schultz *et al*. [50]. However, for antioxidant defense to double lifespan of *daf-2(e1370) *relative to WT animals we would expect to detect substantial decreases of oxidative damage relative to WT worms. We did observe lower levels of carbonylated mitochondrial protein, but only in very old animals. Also, we could not confirm the expected decrease of mtDNA deletion events in the long-lived mutants [51]. In fact, we found no evidence of any such deletions in 624 long-lived and 432 control animals. Moreover, deletion of all mitochondrial SOD activity by null alleles of both MnSOD encoding genes (*sod-2 *and *sod-3*) failed to shorten [52,53] or even extended [54] lifespan in an otherwise wild-type background and failed to abolish longevity of *daf-2(m577) *animals [52]. In all, these and our findings suggest that oxidative damage is not likely a major determinant of the lifespan of *C. elegans *under normal environmental conditions.

### Control of mitochondrial ATP production

We [28,55,56] and others [57] found repeatedly that impairment of IIS signalling resulted in much higher standing levels of ATP than normal. This finding is most surprising as it seems to violate common biochemical wisdom that 'The activities of the pathways that produce ATP are under strict coordinated control so that ATP is never produced more rapidly than necessary' (quoted from [31]). It is not clear which alterations cause this apparent uncoupling in *daf-2 *mutants. The activity of complex V is controlled by the flux of protons and the concentration of ADP in the mitoplasm. Normally, the concentrations of ATP and ADP are in equilibrium: synthesis of ATP is expected to lower the concentration of ADP, in turn lowering the rate of ATP synthesis by complex V. Also, any decrease of the ADP/ATP ratio in the cytosol is expected to result in reduced import of ADP into the mitoplasm tending to maintain the ratio constant. In *daf-2 *mutants both ATP and ADP concentrations are elevated (Figure 1g and 1i), complicating interpretation of their possible role in controlling energy production. Another potential site of control is the cytochrome *c *oxidase (complex IV) reaction which is irreversible. In mammals, ATP is known to bind and inhibit complex IV [58] allosterically, thereby adjusting ATP production to energetic demand. 3,5-diiodothyronine can release the allosteric inhibition of complex IV by ATP, allowing high ATP production in the presence of high concentrations of ATP [59]. Possibly altered IIS signalling in *daf-2 *animals affects an analogous worm control mechanism.

### *daf-2(e1370) *adult animals do not reiterate the energy metabolism of the dauer stage

*daf-2(e1370) *mutant animals inappropriately activate the dauer programme at temperatures (20°-25°C) that allow uninterrupted development of WT worms. Since dauers can survive several times the normal lifespan and *daf-2(e1370) *adults live about twice as long as the WT it is reasonable to expect that they share some common mechanisms for extended lifespan. Whole genome transcription profiling identified a cohort of genes that are upregulated in both dauer larvae and *daf-2(e1370) *adult animals, including genes involved in certain aspects of metabolism, oxidoreductase activity, small heat shock proteins, anti-ROS defense and detoxification systems [25,26,49]. Many of these changes may foster prolonged survival. However, energy metabolism is quite different in dauers and *daf-2(e1370) *adult worms as it is downregulated in dauers and normal in *daf-2(e1370) *adults. Anaerobic fermentation is upregulated and mitochondrial energy production may be partially shifted to anaerobic functioning during dauer diapause, as typically occurs in many parasitic species [24]. The low C/R ratio's measured in *daf-2 *adults exclude similar shifts of energy production in these animals.

### The *daf-2(e1370) *mitochondrial phenotypes are not likely primary mechanisms of *daf-2(e1370) *longevity

The mechanism by which the *daf-2(e1370) *mutation extends lifespan remains elusive. The metabolic profiles of ageing cohorts of N2, *daf-2(e1370) *and *daf-16(mgDf50) *animals are in line with a previous study [30] and, combined, point to a complex regulation of energy metabolism, where two pathways emanate from DAF-2, a predominant one that is DAF-16 independent whereas the other requires DAF-16. Given the common view that DAF-16 is a master regulator of longevity, this would implicate that the metabolic changes imparted by *daf-2(e1370) *are auxiliary, rather than essential, mechanisms of lifespan extension. This view is strengthened by several observations. We have demonstrated that the fall of metabolic rate with age is attenuated in *daf-2(e1370) *animals. Yet, while several aspects of *daf-2(e1370) *mitochondrial function are higher or better preserved with age, state 3 respiration of both WT and *daf-2(e1370) *mitochondria shows no such age-specific fall, suggesting that mitochondrial malfunction is unlikely a primary cause of ageing. Also, the increased energetic efficiency of *daf-2 *animals inferred from the C/R ratio is not recapitulated in isolated mitochondria suggesting that in control of whole-worm metabolism, extra-mitochondrial regulatory mechanisms are important. The higher standing levels of ATP cannot be essential either, because RNAi against several mitochondrial genes reportedly lowered ATP substantially but extended lifespan [57]; also, the *ucp-4(0) *mutant (UCP-4 is the only UCP-like protein encoded in the *C. elegans *genome) contains elevated ATP levels yet is not long-lived [39]. The overproduction of antioxidant enzymes by the *daf-2 *mutant will certainly enhance survival under unfavorable conditions that are associated with oxidative stress, but they appear to be hardly effective in extending lifespan under normal conditions, as discussed previously. Combined, these observations suggest that low *daf-2 *function alters the overall rate of aging by a yet unidentified mechanism, with an indirect protective effect on mitochondrial function.

## Conclusions

We have presented evidence that the age-dependent decrease in abundance and bioenergetic competence is considerably attenuated in mitochondria of the long-lived *daf-2(e1370) *mutant animals, and that these changes are associated with a higher membrane potential and increased ROS production. We also showed that the ultimate mechanism by which the *daf-2(e1370) *mutation extends life span cannot be ascribed to the higher standing levels of ATP or reduced oxidative damage. Thus, the mechanism by which the *e(1370) *mutation extends life span remains largely enigmatic. It is possible though that the altered function of *daf-2(e1370) *mitochondria contributes to shifts in the metabolic network, not detected by the present approach and impinging on longevity assurance mechanisms. It was recently shown that dauer larvae and adult insulin-like signalling (*daf-2*) and translation (*ife-2*) mutants display a common metabolic signature dominated by shifts in carbohydrate and amino acid signature [60]. Many of these metabolites are related to the citric acid cycle, glycolysis, gluconeogenesis and the glyoxylate shunt, metabolic activities that are differently regulated in dauers and *daf-2 *mutant worms. Fuchs *et al*.[60] found a general elevation of amino acid pool sizes in both mutant classes, and a striking upregulation of the branched amino acids isoleucine, leucine and valine, possibly resulting from downregulation of breakdown by mitochondrial BCKD enzyme complex. The upregulation of gluconeogenesis and the glyoxylate shunt and downregulation of amino acids catabolism may serve a longevity assurance mechanism that is based on recycling of cellular components. We assume that dysfunctional mitochondria are more rapidly degraded by autophagic processes in *daf-2(e1370) *mutant animals. The higher standing levels of ATP and the superior bioenergetic competence of their mitochondria would provide the necessary energy for subsequent mitogenesis ensuring sustained presence of competent mitochondria.

## Methods

### Strains, culture and harvest

WT *C. elegans *was the Bristol N2 male stock (Caenorhabditis Genetics Center). Unless otherwise stated, the ins/IGF-1 pathway mutants used were: *daf-2(e1370) *and *daf-16(mgDf50)*.

Up-scaling, synchronization and metabolic rate determination of nematode cultures was essentially as described previously [28,61].

The animals were cultured on agar plates seeded with *Escherichia coli *K12 at 17°C to by-pass dauer formation in *daf-2(e1370)*. When the total population size needed for all successive harvests was reached (approximately 5 million worms), the nematodes were rinsed off the plates shortly after the molt to the fourth larval stage, suspended in S-buffer containing 100 μM FUdR (to suppress reproduction) at densities not exceeding 2000/ml and *E. coli *cells were added at ~3 × 10^9 ^cells/mL. Two hundred and fifty millilitre portions were transferred into Fernbach flasks that were placed in a gyrotory shaker incubator (New Brunswick Scientific, NJ, USA) and shaken at 120rpm at 24°C. To maintain the initial concentration of bacteria, turbidity at 550nm was measured daily and a concentrated *E. coli *suspension was added as needed. Harvesting started approximately 24 h after the worms were shifted from 17° to 24°C; we defined this time point as day 0 of adulthood. Samples containing approximately 300,000 live worms were harvested at regular intervals and freed from dead worms, bacteria and debris as described [61]. The cleaned worms were suspended in 15 mL S-buffer and aliquots of 0.1 mL were pipetted into microcentrifuge tubes and stored at - 75°C for assays that can be performed using frozen worms (ATP and ADP content, citrate synthase activity, cytochrome *c *Western Blot, protein determination). For assays that require live animals, an aliquot containing 900 μL worms was transferred to a 15mL tube and diluted to 5 mL with basal axenic medium (3% soy peptone and 3% yeast extract). From this suspension, 1ml aliquots were used for oxygen consumption and heat dissipation assays. The bulk of live animals were used for the isolation of mitochondria.

### Metabolic rate measurements, and quantification of ATP and ADP

Respiration and heat dissipation rates by living animals were performed and corrected for body volume as previously described [28,61]. ATP and ADP were extracted from frozen worm tissue with perchloric acid as follows: to 100 μL of nematode suspension in S-buffer, 400 μL of HClO_4 _8% (v/v) and 200 mg of glass beads were added. The samples were homogenized using a Mini-Beadbeater (Biospec Products, OK, USA), operated at 5000 strokes/min for 1 min. Next, 450 μL (three consecutive portions of 150 μL to avoid excessive formation of bubbles) of 1.33 M KHCO_3 _and 150 μL H_2_O were added. After leaving for 15 min at room temperature the sample was degassed in a Savant Speed Vac Concentrator for 10 min and cleared by centrifugation at 14000 rpm for 8 min. Aliquots of the supernatant were used for ATP determination using the ATP Bioluminescence Assay Kit CLS II (Roche Diagnostics, Mannheim, Germany) and a Wallac Victor^2 ^Multilabel Counter (Perkin-Elmer, MA, USA) as previously described [61]. ADP was measured as excess ATP detected after conversion of all ADP to ATP. This was achieved in a coupled reaction in which 64 μM phosphoenolpyruvate was converted to pyruvate in 40 mM potassium phosphate, pH 7.6 in the presence of 4 mM MgSO_4 _and 1 U/mL pyruvate kinase. After leaving for 10 min at room temperature, the reaction was stopped by heating the samples for 8 min at 99°C, and the supernatant was cleared by centrifugation at 14000 rpm for 8 min. Total ATP determination was performed as described and the ADP content was calculated as the difference between total ATP and ATP content before conversion of ADP.

### Citrate synthase activity in crude extract and mitochondria

Mitochondrial preparations (prepared as described in section 'preparation and handling of mitochondria') were made 1% in CHAPS and the resulting solution was clarified by centrifugation at 14,000 rpm for 10 min. Whole-worm extract was prepared as described by [61]. Citrate synthase was assayed by monitoring the reduction of 5,5'-dithiobis(2-nitrobenzoic acid; DTNB) at 412nm (ε_412 _= 13.6 mM^-1 ^cm^-1^) coupled to the reduction of Coenzyme A by the citrate synthase reaction in the presence of oxaloacetate. The protocol described by [62] was adapted for use with microtitre plates. Briefly, 0.1 M Tris-HCl (pH 8.0), 0.3mM acetyl-Coenzyme A, 0.1mM DTNB, and samples of the mitochondrial preparation containing approximately 5 μg protein or whole-worm extract containing approximately 40 μg protein were incubated for 10 min at 24°C. The reaction was initiated by the addition of 0.75 mM oxaloacetate (final concentration) and the rise in absorbance at 412 nm was monitored for 3 min in a Spectramax 190 (Molecular Devices, CA, USA) plate reader.

### Preparation and handling of mitochondria

Mitochondria were isolated essentially following the method described by [63]. Briefly, approximately 300,000 age-synchronized animals were harvested from the cultures at regular time intervals, cleaned and washed with distilled water to remove the S buffer and finally suspended in mitochondrial isolation buffer (MSME) according to [63]. All subsequent treatments were performed at 4°C. The worm suspension was transferred to a sampling tube and chopped, using an IKA Ultraturrax rotor-stator mixer (IKA Werke, Staufen, Germany). Next, an equal volume of MSME containing 0.4% bovine serum albumin (BSA) was added and the suspension was thoroughly mixed by inversion, and centrifuged for 5 min at 380g to remove large debris and nuclei. The supernatant was transferred to a fresh centrifuge tube and centrifugation was repeated. The resulting supernatant containing crude mitochondria was centrifuged for 5 min at 4500g and the resulting mitochondrial pellet was resuspended in MSME. Aliquots were used for instant measurement of mitochondrial respiration, ATP synthesis, membrane potential and production of H_2_O_2_. The remainder was frozen at -75°C in 10 μL aliquots for quantification of citrate synthase activity, cytochrome *c *and carbonylation levels and protein content. On average, 300,000 nematodes yielded ~1mg of mitochondrial protein.

### Oxidative phosphorylation measurements

Oxygen consumption by isolated mitochondria was monitored polarographically using a Clark type electrode mounted in a respirometer cell and connected to an oxygen meter (Strathkelvin Mitocell MT200A and 782 Single/dual channel oxygen meter, Strathkelvin Instruments, Glasgow, Scotland). An aliquot of isolated mitochondria containing approximately 300 μg of protein was added to 500 μL of air saturated incubation medium [63] at 24°C. Respiration was activated by adding 5 mM pyruvate and 5 mM malate (final concentrations) followed by sequential additions of 50 nmol ADP. State 3 and state 4 oxygen consumption, respiratory control ratio (RCR) and ADP/O were calculated according to (32). Oxygen consumption in the presence of uncoupler was measured by adding 5 μM of FCCP.

### Quantification of ATP synthesis

ATP synthesis was determined using the Roche ATP Bioluminescence Assay Kit CLS II. Approximately 5 ng of freshly isolated mitochondria were added to the wells of a white microtitre plate containing 96 μL of incubation medium [63], 50 μL of luciferase reagent [one bottle was dissolved in 5 mL of sterile high-performance liquid chromatography (HPLC) water] and 50 μL of substrate/ADP mix (final concentrations in the well: pyruvate 1 mM, malate 1 mM, ADP 100 μM). The emitted light was measured for 45 min in a Wallac Victor^2 ^Multilabel Counter. For determination of background light emission, 0.4 μg oligomycin was added.

### Mitochondrial heat dissipation and calorimetric-to-respirometric ratio

For this assay, mitochondria were isolated in the presence of a protease inhibitor cocktail (Roche Diagnostics) at the concentration recommended by the manufacturer. Heat dissipation was registered by the Thermal Activity Monitor (TAM, TA Instruments, DE, USA) as follows: 560 μL of phosphate-enriched incubation medium (100 mM KCl, 50 mM MOPS, 1 mM EGTA, 100 mM potassium phosphate, 1 mg/mL defatted BSA, pH7.4) was transferred to a glass ampoule and made 27.4 mM each in pyruvate and malate from pH 7 stock solutions. Next, ADP and penicillin/streptomycin mixture were added at 16.4 mM and 200 units/200 μg, respectively. Protease inhibitor cocktail was added as needed to meet the recommended concentration. Finally, 250-500 μg of mitochondria were added and the ampoule was sealed and inserted into the TAM. An identical sample was taken concurrently for registration of a state 3 oxygen consumption rate. Heat dissipation was recorded for 10 min after approx. 40 min of equilibration. Immediately following termination of the recording, the contents of the ampoule were transferred to the cell of the respirometer and a state 3 oxygen consumption rate was determined. This second reading was used for obtaining the mitochondrial C/R ratio.

### Determination of the membrane potential

For this assay, mitochondria were isolated in the presence of a protease inhibitor cocktail (Roche Diagnostics) at the concentration recommended by the manufacturer. The fluorescent probe DASPMI (dimethylaminostyrylmethylpyridinium-iodine) was kindly provided by Professor Jürgen Bereiter-Hahn and used as an indicator of mitochondrial membrane potential, essentially following the protocol by [32], with minor changes. Protein concentration in the mitochondrial preparations was determined according to [64] and adjusted as needed to obtain a ratio of approx. 2.9 nmol DASPMI/mg mitochondrial protein in the sample wells. Briefly, to a well of a black microtitre plate, 234 μL of incubation medium was added, followed by 3 μL of a 1M succinate stock, 3 μL of a 400 μg/mL rotenone solution in DMSO and 15 μL of a 96 μM DASPMI solution in HPLC-grade water. Next, 30 μl of mitochondrial suspension was added and fluorescence was recorded (Wallac Victor^2 ^Multilabel Counter, excitation at 450 nm, emission at 590 nm) for 4 min to obtain a stable mitochondrial membrane potential signal. Next, 4 μL of a 200mM ADP stock solution was added and DASPMI fluorescence in energized mitochondria was recorded for 2 min. Finally, the membrane potential of uncoupled mitochondria was registered for 2 min after addition of 10 μL of a 1 mM FCCP solution. Fluorescence signals were corrected for small differences in protein content using the BCA (bicinchoninic acid) method (see section '*Western blotting, carbonylation assay and protein determination'*) which is more sensitive than the Bradford assay.

### Quantification of mitochondrial H_2_O_2 _formation

Mitochondrial H_2_O_2 _production was measured according to standard procedures by the horseradish-peroxidase-mediated oxidation of Amplex Red (Invitrogen, CA, USA) to the fluorescent compound resorufin. Freshly isolated mitochondria were incubated with the appropriate substrates and ADP and the rate of H_2_O_2 _production was measured with Amplex red as follows: aliquots of 96 μl incubation medium containing 4 mM ADP, 10 mM of pyruvate, 10 mM of malate and 10 U of Cu/ZnSOD (from bovine erythrocytes) were added to the wells of a black microtitre plate. The final assay medium was obtained by adding 100 μL of a mixture containing 100 μM of Amplex Red and 4 U/mL of horseradish peroxidase. Next, approximately 20 μg of freshly isolated mitochondria were added and the emitted fluorescence was measured for 35 min in the Wallac Victor^2 ^Multilabel Counter, at excitation and emission wavelengths of 550 and 590 nm, respectively. The intensity of fluorescence was converted to picomoles of H_2_O_2 _by running an internal H_2_O_2 _standard curve.

### Western blotting, carbonylation assay and protein determination

Protein was generally determined using the BCA method as described [65] but prior degradation with alkali was only applied for estimating whole worm protein content. The protein content of samples dissolved in Laemmli buffer was measured using the Pierce 660 nm protein assay (Thermo Scientific, IL, USA). For quantification of cytochrome *c *in whole worm extract and in mitochondrial preparations, frozen samples with known protein concentration were mixed with Laemmli buffer, heated at 99°C for 5 min and equal amounts of protein were loaded on gels. For all other antibodies, live worms were lysed directly with Laemmli buffer and stored at -80°C. Prior to Western blotting, samples were thawed, protein concentration was determined and equal amounts of protein were loaded and run. Western blotting was performed as described by [66]. Primary antibodies against cytochrome *c*, complex I NDUFS3 subunit, pyruvate dehydrogenase subunit E1alpha, complex IV subunit I, complex V subunits alpha and beta and adenine nucleotide transferase were purchased from Mitosciences (OR, USA).

Secondary antibody was horseradish peroxidase (HRP)-conjugated anti-mouse antibody from Sigma.

The carbonyl load of mitochondrial protein was measured using a Western immunoblot assay after protein derivatization with DNPH, as previously described [67].

### DNA isolation and amplification

We scanned the long-lived strain *daf-2(e1370); glp-4(bn2) *and the control strain *daf-16(mgDf50) glp-4(bn2) *for possible deletions in mtDNA. Age-synchronized L1 larvae were grown on nutrient agar plates seeded with *E. coli *OP50 at 17 °C until they reached the third juvenile stage to avoid induction of dauer formation at higher temperature by the mutation in *daf-2*. Next they were shifted to 24°C to induce the germline defective phenotype caused by mutation in *glp-4*. For nucleic acid extraction, 3-, 7- and 10-day-old adult *daf-16(mgDf50) glp-4(bn2) *worms and 3-, 7-, 10-, 14- and 26-day-old *daf-2(e1370); glp-4(bn2) *worms were transferred to individual microcentrifuge tubes containing 25 μL of worm lysis buffer (50 mM KCl, 2.5 mM MgCl_2_, 0.15% NP40, 0.15% Tween 20, 10 mM Tris.HCl pH 8.3) and frozen at -75 C for 10 min. Next, the samples were thawed and 1 μL of proteinase K was added at 100 μg/μL final concentration and protein was digested for 1 h at 65 °C, followed by 10 min at 95 °C to inactivate the enzyme. These samples were used for nested PCR without any further purification. One microlitre sample and 0.25 μL TaKaRa LA Taq (TaKaRa Bio Inc, Shiga, Japan) were added to PCR tubes containing 24 μL of PCR components and the outer pair of primers (Forward: CTTGTTCCAGAATAATCGGCTAGACTTGTTAAAGCTTGTAC, reverse: CCTAAGCCCTAGGCCCAAAGTAACTATTGAAAAACC), and subjected to 25 cycles (30 s at 94°C, 30 s at 60°C and 12 min at 68°C) of PCR to generate a fragment of 11,492 bp. Next 1 μL of this mixture was transferred to a tube containing 24 μL fresh reaction components and the inner pair of nested primers (forward: GGAGGCTGAGTAGTAACTGAGAACCCTC, reverse: GTGAAAGTGTCCTCAAGGCTACCACCTTC) to generate a final PCR fragment 11,211 base pairs long. As a control experiment, we also used the Melov primers for the second PCR reaction, generating a 6294 bp long fragment [51]. The amplified DNA fragments were analyzed on 0.7% agarose gel. We found that fragments larger than ~7 kb would not be resolved from the full-length (~11 kb) amplicons. As a positive control, *him-8(e1489); uaDf5/+ *, a strain heteroplasmic for a 3.1-kb deletion in mtDNA [68], did result in both 6kb amplicons and shorter amplicons of ~3kb when the second set of primers were used.

### Determination of mtDNA copy number

mtDNA copy number was assayed according to [69]. A standard curve for determining the mitochondrial genome content was obtained as follows. Staged worms were lysed as described and PCR was performed to amplify 3 mitochondrial genes: ND5 (forward primer: CCACACCGGTGAGGTCTTTGGTTCATAGTAG; reverse: GTGAAAGTGTCCTCAAGGCTACCACCTTC), COII (forward: TCGTTGTGTTATTCCTTGTGATACT, reverse: ACAAATCTCTGAACATTGACCATAA) and COIII (forward: TACAGTAACTTGAGCACATCACAGA, reverse: ATACTCCGTCTGCAATAGAAAATCT)

The PCR product was purified with the QIAquick PCR purification kit (Qiagen, Venlo, The Netherlands) and the concentration of the resulting templates was determined using the NanoDrop ND-1000 diode array spectrophotometer (NanoDrop Technologies, DE, USA). The copy number was then calculated from the weight in Daltons and Avogadro's number and a serial dilution was used to generate a standard curve for quantitative PCR.

Increasing amounts of template (10-10^8 ^copies per reaction) were amplified and the standard curve was constructed by plotting the cycle threshold (Ct) values versus the logarithm of the initial template copy number. For assessing the average mitochondrial genome content per worm we grew parallel cultures of N2 and *daf-2(e1370) *and harvested staged worms of increasing age. This scheme was repeated three times to account for environmental variation. DNA samples were prepared from 48 single worms per time point and pooled to dampen variation in individual mitochondrial genome content. QPCR amplification was carried out using the Qiagen Rotor-Gene real-time cycler with Invitrogen Platinum SYBR Green qPCR SuperMix-UDG. The cycling conditions were as follows: 50°C for 2 min, initial denaturation at 95°C for 2 min followed by 45 cycles of 15 s at 95°C, 30 s at 60°C and 30 s at 72°C. Following the final cycle, melting curve analysis was performed to examine the specificity in each reaction tube (absence of primer dimers and other non-specific products). We first determined the mtDNA content as a function of age and strain differences and found that all three amplicons yielded moderately diverging estimates of the mitochondrial copy number but with equal age- and strain-related trends. Absolute copy numbers were therefore normalized as follows: for each primer pair, wild-type values from all ages were averaged and this value was set to 100. All original values were then rescaled relative to this reference value and pooled to obtain an estimate of mtDNA content per animal relative to the reference value.

### Statistical analyses

Regression analysis of age-related changes was performed using the mixed linear regression model PROC MIXED in SAS statistical software; data were log-transformed when needed to allow the best possible fit and tests of fixed effects provided *p *values for strain, age and age_*_strain. When *P*_age*strain _was < 0.05, age-related changes (slopes) differed significantly between the strains compared. When *P*_age*strain _was not significant, further conclusions were drawn based on *P*_age_, *P*_strain _or both. As an auxiliary analysis, a Student's *t*-test was used to compare differences between strains or ages at specific time points. Error bars depict standard error of the mean.

### Abbreviations

ADP: adenosine diphosphate; ATP: adenosine triphosphate; BSA: bovine serum albumin; CHAPS: 3-[(3-cholamidopropyl)dimethylammonio]-1-propanesulfonate; C/R: calorimetric to respirometric; ct: cycle threshold; *ctl*: *c*a*t*a*l*ase; Cu/ZnSOD: copper/zinc superoxide dismutase; *daf *: abnormal *da*uer *f*ormation; DMSO: dimethyl sulphoxide; DNPH: 2,4-dinitrophenylhydrazine; EGTA: ethylene glycol-bis(2-aminoethylether)-N,N,N',N'-tetraacetic acid; FCCP: carbonyl cyanide 4-(trifluoromethoxy)phenylhydrazone; FOXO: Forkhead Box O; FUdR: 5-fluoro-2'-deoxyuridine; *glp *: abnormal *g*erm *l*ine *p*roliferation; *gst *: *g*lutathione *S*-*t*ransferase; *him *: *h*igh *i*ncidence of *m*ales; HRP: horseradish peroxidase; *hsp *: *h*eat *s*hock *p*rotein; IGF-1: insulin growth factor-1; MnSOD: manganese superoxide dismutase; *mtl *: *m*e*t*a*l*lothionein; mtDNA: mitochondrial DNA; NS: not significant; OXPHOS: oxidative phosphorylation; PCR: polymerase chain reaction; RCR: respiratory control ratio; ROS: reactive oxygen species; SDS-PAGE: sodium dodecyl sulphate polyacrylamide gel electrophoresis; SOD: superoxide dismutase; MOPS: 3-(N-Morpholino) propanesulphonic acid; *sod *: *s*uper*o*xide *d*ismutase; UCP: uncoupling protein; WT: wild-type; ΔΨ_mit _: mitochondrial membrane potential.

### Authors' contributions

KB designed and performed most of the experiments, analysed most of the data and co-wrote the manuscript. NC performed some of the experiments. FM performed some of the experiments. JRV designed the study and co-wrote the manuscript. BPB designed the study and co-wrote the manuscript.

## Supplementary Material

Additional file 1 **The *daf-2(e1370) *allele attenuates the age-specific decline in abundance of key mitochondrial proteins**.
(A-F) Western blots showing age-related changes in the abundance of important mitochondrial proteins in crude worm extract. The numeric values for each protein denote its abundance in 1-,3-,6-,9- and 12-day-old adults normalized to the abundance in 1-day-old wild-type adults and are plotted in the corresponding graphs.
(A) Complex I NDUFS3 subunit. (B) Pyruvate dehydrogenase subunit E1 alpha.
(C) Complex IV subunit I. (D) Complex V subunit alpha. (E) Complex V subunit beta. (F) Adenine nucleotide transferase. Click here for file

Additional file 2 **C/R ratios of mitochondria isolated from wild-type and *daf-2(e1370) *mutant worms**. Mitochondria were isolated from 2-day-old adults and fuelled with Complex I substrates and adenosine diphosphate to activate complex-I-dependent respiration for at least 1 h. The oxygen consumption rates measured after completion of calorimetry were used for calculating the mitochondrial calorimetric to respirometric ratio. Data represent means ± standard error of mean for mitochondria isolated from eight replicate cultures. Click here for file

Additional file 3 **Supplemental Figure S3 - Loss of DAF-16 activity does not affect either H_2_O_2 _production by wild-type mitochondria nor the carbonyl load of mitochondrial protein**. Data represent means ± standard error of mean for mitochondria isolated from three replicate cultures. Click here for file
