# Supplementary figures and images for: Disruption of insulin signalling preserves bioenergetic competence of mitochondria in ageing Caenorhabditis elegans
Source: BMC Biol. 2010 Jun 28;8:91. doi: 10.1186/1741-7007-8-91 (PMC2914644; doi:10.1186/1741-7007-8-91)

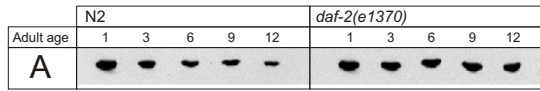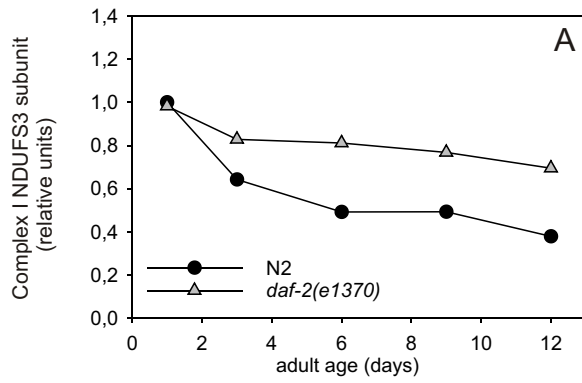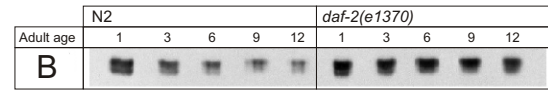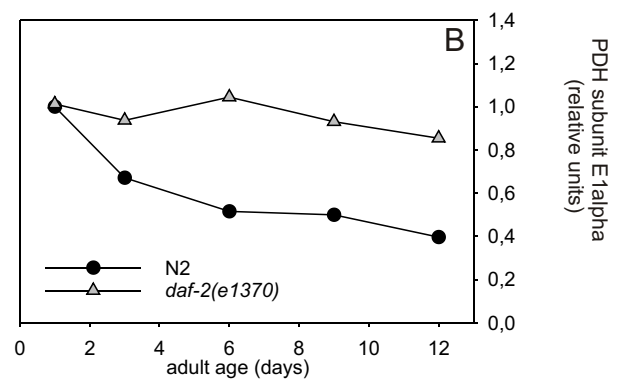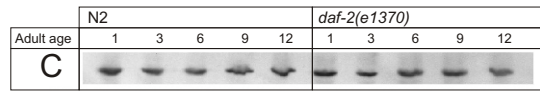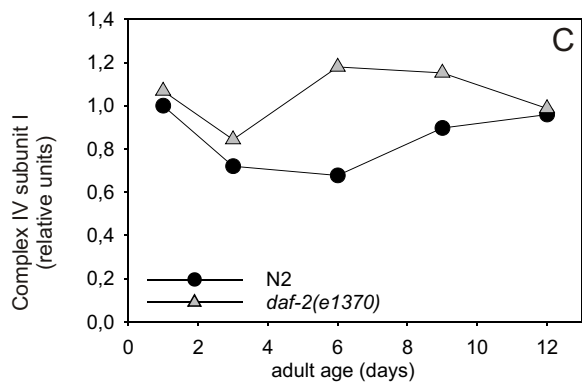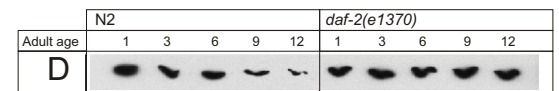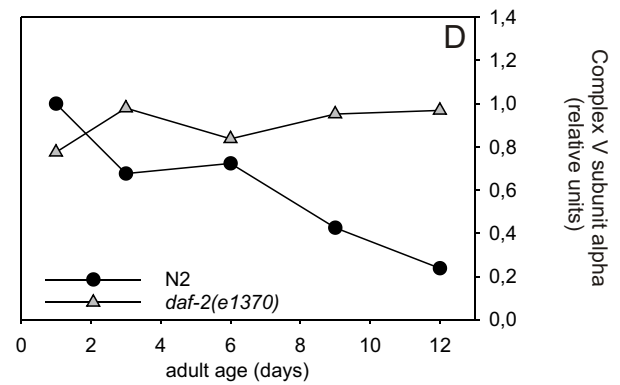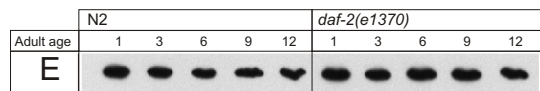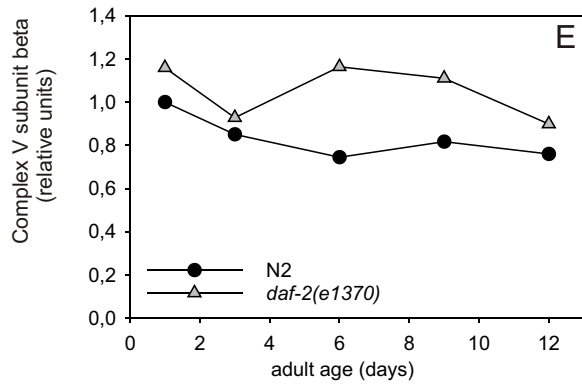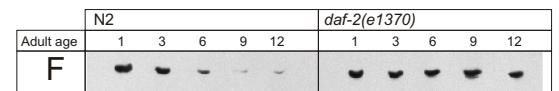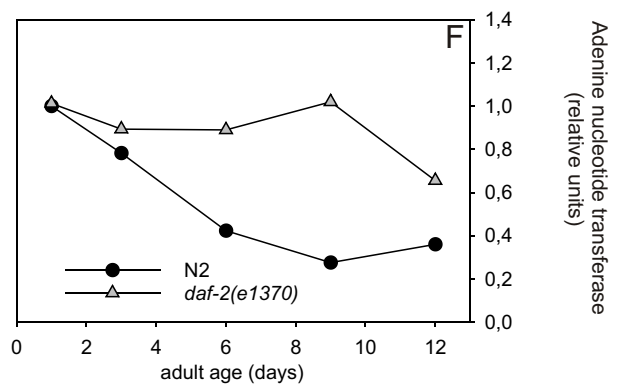

Supplement: Additional file 1 — The daf-2(e1370) allele attenuates the age-specific decline in abundance of key mitochondrial proteins. (A-F) Western blots showing age-related changes in the abundance of important mitochondrial proteins in crude worm extract. The numeric values for each protein denote its abundance in 1-,3-,6-,9- and 12-day-old adults normalized to the abundance in 1-day-old wild-type adults and are plotted in the corresponding graphs. (A) Complex I NDUFS3 subunit. (B) Pyruvate dehydrogenase subunit E1 alpha. (C) Complex IV subunit I. (D) Complex V subunit alpha. (E) Complex V subunit beta. (F) Adenine nucleotide transferase. [file 1741-7007-8-91-S1.PDF]

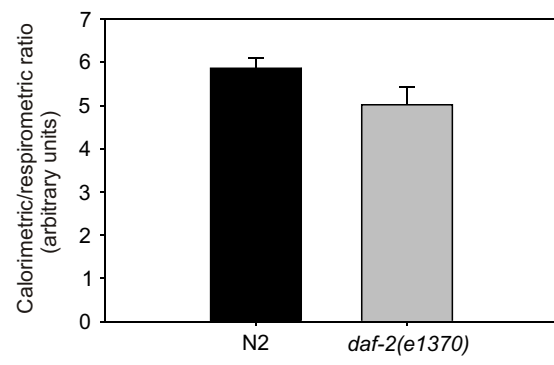

Supplement: Additional file 2 — C/R ratios of mitochondria isolated from wild-type and daf-2(e1370) mutant worms. Mitochondria were isolated from 2-day-old adults and fuelled with Complex I substrates and adenosine diphosphate to activate complex-I-dependent respiration for at least 1 h. The oxygen consumption rates measured after completion of calorimetry were used for calculating the mitochondrial calorimetric to respirometric ratio. Data represent means ± standard error of mean for mitochondria isolated from eight replicate cultures. [file 1741-7007-8-91-S2.PDF]

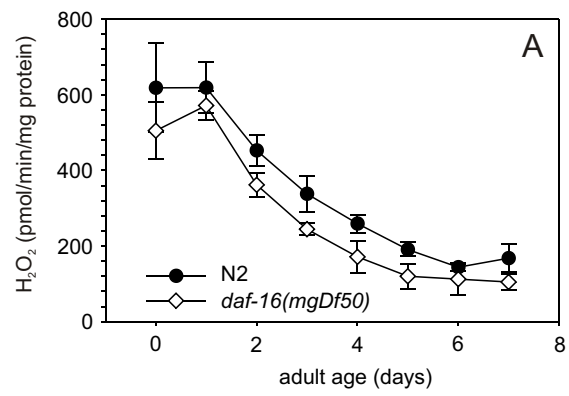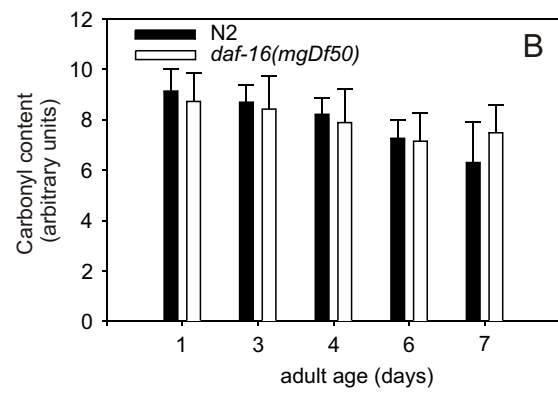

Supplement: Additional file 3 — Supplemental Figure S3 - Loss of DAF-16 activity does not affect either H2O2 production by wild-type mitochondria nor the carbonyl load of mitochondrial protein. Data represent means ± standard error of mean for mitochondria isolated from three replicate cultures. [file 1741-7007-8-91-S3.PDF]
